# Scanning the horizon of personalized prevention research: an overview of ongoing European funded initiatives

**DOI:** 10.3389/fpubh.2025.1561328

**Published:** 2025-08-12

**Authors:** Alessandra Maio, Sara Farina, Tommaso Osti, Salvatore Di Grande, Roberta Pastorino, Stefania Boccia

**Affiliations:** ^1^University Department of Life Science and Public Health, Section of Hygiene, Università Cattolica del Sacro Cuore, Rome, Italy; ^2^Department of Woman and Child Health and Public Health, Fondazione Policlinico Universitario A. Gemelli IRCCS, Rome, Italy

**Keywords:** personalized prevention, research, non-communicable diseases, European Commission, projects

## Abstract

**Introduction:**

Non-communicable diseases represent a significant burden on global healthcare, necessitating innovative strategies to enhance prevention and management. Personalized prevention, an emerging approach leveraging omics data to tailor interventions, holds promise for improving risk stratification, early diagnosis, and preventive strategies. To gain insights on the latest funding investments in this field, we mapped European Commission (EC)-funded research projects on personalized prevention for non-communicable diseases.

**Materials and methods:**

We carried out a scoping review of gray literature sources, following the Arksey and O’Malley framework, combined with expert consultations to validate findings and address data gaps. Inclusion criteria focused on projects that began or were ongoing in 2024 (from January 1st to December 31st).

**Results:**

We identified 67 projects supported by a total amount of €511.9 million from EC funding. The main programs include Horizon Europe, Horizon 2020, and EU4Health. In particular, Horizon Europe funds 51% of these projects, with a total allocation of €253.8 million, 44% of which specifically address cancer. Overall, 48% of the projects target cancer, followed by neurological and psychiatric diseases (15%), cardiovascular diseases (13%), metabolic disorders (9%), and other NCDs (16%). In terms of prevention levels, 21% of the projects are dedicated to primary prevention, 41% to secondary prevention, and 38% to tertiary prevention.

**Conclusion:**

The EC’s investment in personalized prevention is predominantly directed toward cancer, reflecting the goals of the Cancer Mission and the European Beating Cancer Plan. Research on tertiary prevention remains less prominent, likely due to its already established clinical applications, while the emphasis on primary and secondary prevention is reassuring, considering the current gaps in clinical utility evidence in these areas.

## 1 Introduction

Non-communicable diseases (NCDs) represent a significant global challenge for healthcare systems, requiring long-term management, substantial resource allocation, and adversely affecting patients’ quality of life. These conditions arise from a complex interplay of genetic, environmental, and lifestyle factors, making their management particularly challenging. Such complexity necessitates the development of increasingly tailored and adaptive approaches that account for the unique characteristics of individual patients (1).

In recent years, personalized medicine has emerged as a transformative paradigm in healthcare. By customizing interventions to the unique characteristics of each patient, personalized medicine marks a departure from the traditional “one-size-fits-all” model, extending its application not only to treatment but also to the prevention of diseases (2). The integration of this concept with classical paradigms of prevention opens up a new approach, which is called personalized prevention. Through the integrated use of “omics,” that refers to the large-scale, comprehensive study of classes of biological molecules, such as genes (genomics), proteins (proteomics), or others, using high-throughput technologies to analyze and quantify all members of a given molecular category within a biological system (3), personalized prevention can in principle improve risk stratification, facilitate early diagnosis, and support tailored preventive interventions (4). Despite the promising potential there are still many barriers to the adoption of personalized prevention approaches: from evidence in terms of clinical utility to evidence in the areas of sustainability and acceptability. For instance, while polygenic risk scores (PRS) offer promising insights, because of the ability to accurately reclassify disease risk, the actual impact in improving health outcomes has yet to be substantiated in large-scale clinical settings, largely due to a lack of rigorous prospective research (5). Additionally, critical factors such as the transferability of evidences into broad racial and ethnic groups and different health systems remain underexplored, underscoring the need for more representative studies in personalized prevention research (6) In response to these challenges, the project titled “a *PeRsOnalized Prevention roadmap for the future HEalThcare”* (PROPHET) (7, 8) funded as Coordinating and Support Action of the International Consortium of Personalized Medicine (ICPerMed) (9) by the European Union (EU)‘s Horizon Europe program, aims to facilitate the adoption of personalized prevention as a mainstream approach within healthcare systems. According to the PROPHET consortium, personalized prevention aims to prevent onset, progression and recurrence of diseases through the adoption of targeted interventions that consider the biological information (e.g., genetics and other biomarkers), environmental and behavioral characteristics, socio-economic and cultural context of individuals. This should be timely, effective and equitable in order to maintain the best possible balance in lifetime health trajectory (7, 8). In this context, our work aims to provide a comprehensive overview of ongoing European Commission (EC)-supported research in personalized prevention toward NCDs. By reporting a complete snapshot of EC investments in this area, our study seeks to identify current research priorities and highlight areas where further investment and scientific focus are needed to advance the clinical implementation of personalized prevention approaches.

## 2 Materials and methods

The main objective of this study was to map ongoing EC-funded research projects on personalized prevention, defined by the PROPHET consortium as “a set of interrelated activities aimed at achieving deliverables or outputs aligned with the research program funding them.”

To achieve this objective, a two-stage methodological process was implemented:

1. Phase 1: desk research;
2. Phase 2: expert consultation.

The full protocol is publicly accessible on the Open Science Framework (DOI: 10.17605/OSF.IO/HBZJ7).

### 2.1 Desk research

We conducted a scoping review according to the methodology established by Arksey and O’Malley (10), with the PRISMA-ScR (Preferred Reporting Items for Systematic Reviews and Meta-Analyses extension for Scoping Reviews) Checklist applied to ensure adherence to reporting standards (11).

#### 2.1.1 Search strategy

We collected documents using gray literature sources, focusing on the Community Research and Development Information Service (CORDIS) (12)—the official portal of the EC for disseminating information on EU-funded research and innovation projects—and the EC’s website (13). To identify relevant materials, we developed a search strategy structured around the general term “project,” aimed at retrieving records related to EU-funded research initiatives, and a cluster focused on “personalized prevention” and synonyms, in order to capture the diverse terminology used to describe this evolving field across different projects and funding schemes.

The adapted string we applied was the following: (project*) AND (“personal* prevention” OR “individual* prevention” OR “predictive prevention” OR “precision prevention” OR “stratified prevention” OR “tailored prevention”).

We deliberately chose not to include terms related to specific chronic diseases, to maintain a broad and sensitive search strategy and avoid missing relevant projects that address personalized prevention across various health domains, even when not explicitly labeled by disease.

#### 2.1.2 Eligibility criteria

To be included, records had to meet the following criteria: they must represent a research project funded or co-funded by the EC, focus on personalized prevention, and be related to NCDs. Only projects that started or were ongoing in 2024 (between January 1st and December 31st) were considered, and the documentation had to be available in English. Records related to deliverables, publications, or public summaries were excluded. Additionally, projects were excluded if they did not focus on prevention or omics sciences, and if they addressed communicable diseases. Projects completed before 2024 or with documentation not available in English were also excluded.

The records identified through the search were subjected to a two-stage screening process. Given that gray literature often lacks abstracts, the initial screening was based on the document’s title and main subject (e.g., project technical sheets, program descriptions, deliverables, publications, or public summaries). A second, full-text screening was then performed. Both stages were conducted independently by two researchers to ensure consistency and a third researcher to resolve any disagreements that arose during the evaluation process.

#### 2.1.3 Data charting

For each eligible project, we extracted on the title and acronym, Grant Agreement ID, start and end dates, coordinating entity (including name, country, and type of activity), funding program, funding amount, project website, type of disease addressed, level of prevention investigated (e.g., primary, secondary, tertiary), and general objective. Data extraction was independently conducted by researchers to maintain reliability.

### 2.2 Expert consultation

All professionals belonging to the PROPHET consortium and its advisory board were included in the expert panel, given their specific expertise in the field of personalized prevention. Their selection was further justified by the considerable heterogeneity in terms of professional background, institutional affiliation (including academic institutions, public bodies, and private organizations), and geographical distribution across multiple European countries. Each expert received a technical sheet via email, listing the projects identified through desk research and all the information extracted. Their feedback was used to validate the list and fill in missing information from the official documents in their possession, as data on CORDIS is often incomplete (14). Experts were also asked to identify any additional relevant projects by completing a dedicated form. Two researchers subsequently verified that the projects submitted through this form met the inclusion criteria. Data collection was concluded in October 2024. This strategic approach enhanced both the efficiency and the completeness of the desk research data, improving the overall quality and relevance of the information on ongoing research projects.

### 2.3 Data synthesis

We reported a qualitative description of the variables extracted according to the specific funding program. This approach facilitated a structured comparison of the disease types targeted by the projects, and the levels of prevention addressed, thus emphasizing the contributions and primary focus areas of each funding program.

## 3 Results

**Figure 1:**
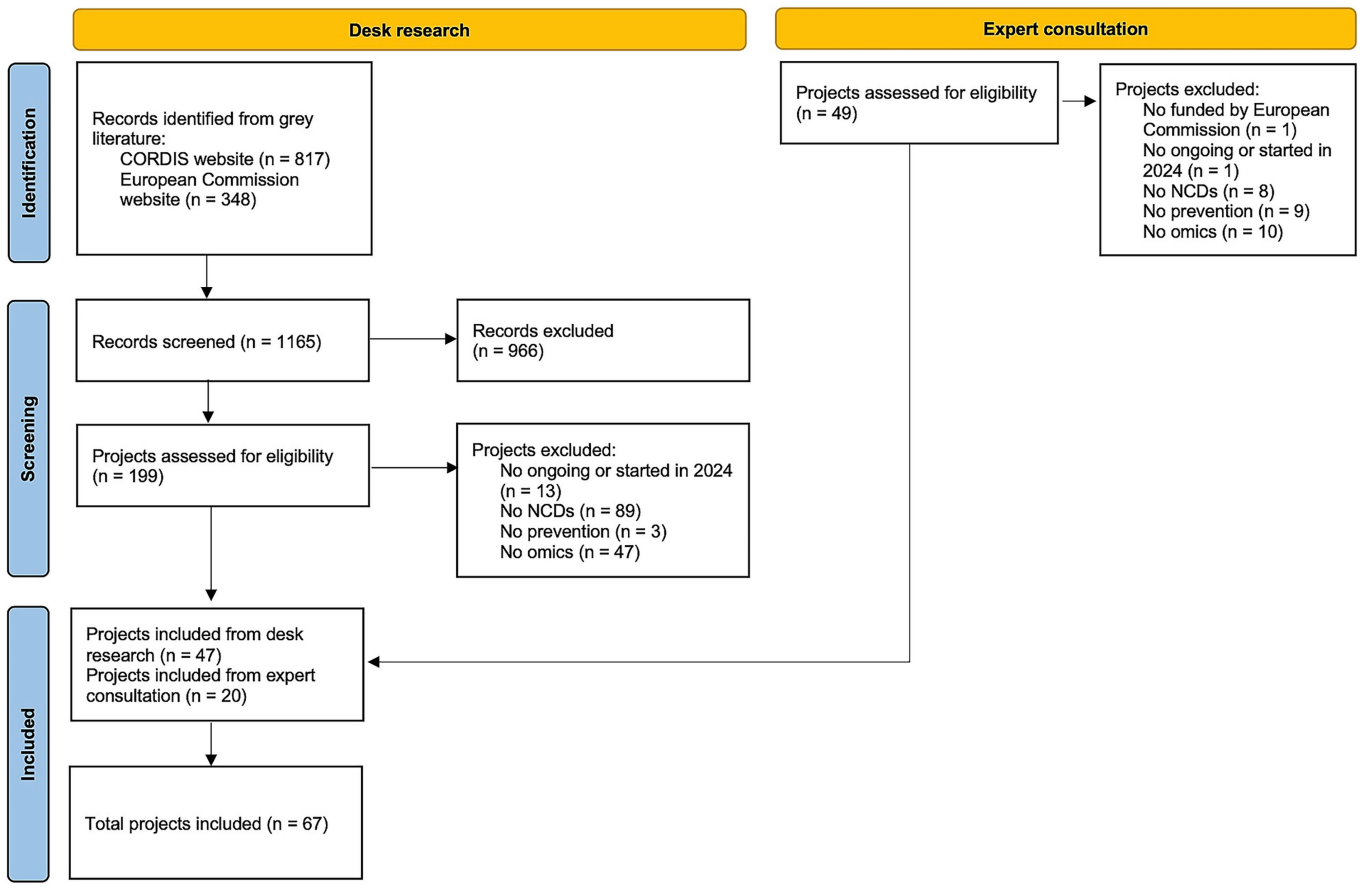
PRISMA flowchart for study selection.

A total of 67 research projects were included (15–81), of which 47 derived from the desk research, and 20 from the expert consultation, as shown in the flowchart (Figure 1). Full projects characteristics are reported in Supplementary material S1.

### 3.1 Project characteristics

In terms of project timelines, the projects started between 2017 and 2024, with expected completion dates ranging from 2024 to 2033. Project durations vary from 1 to 10 years, with a median duration of 4 years (interquartile range: 3–5 years). The total budget allocated by the EC for personalized prevention projects amounts to € 511,935,689.10.

A summary of the main characteristics of the included projects is presented in Table 1. Regarding project coordination, 42% of the projects are led by higher or secondary educational institutions, followed by 34% coordinated by research organizations, and 7% by public organizations. The remaining 17% of projects are coordinated by various private and non-private entities. Twenty-one countries were identified as coordinators of at least one project funded by the EC, including 16 from the EU countries and 5 from non-EU countries. Italy leads with 15% of the projects, followed by the Netherlands (11%), France (10%), and Germany and Belgium (each 9%).

**Table 1:** Description of the EU-funded projects on personalized prevention according to selected variables.

Total projects	(*N* = 67)
Projects characteristics	*N* (%)
Coordinator	
**Type of entity**	
Higher or secondary educational institutions	28 (42)
Research organizations	23 (34)
Public organizations	5 (7)
Other	3 (5)
Public bodies (excluding research organizations and secondary or higher education establishments)	2 (3)
Private for-profit entities (excluding higher or secondary education establishments)	2 (3)
Private non-profit research organizations	2 (3)
Privates small and medium-sized enterprises	1 (1)
Small or medium-size enterprises	1 (1)
**Country**	
Italy	10 (15)
Netherlands	8 (11)
France	7 (10)
Germany	6 (9)
Belgium	6 (9)
Spain	5 (7)
Greece	4 (6)
Finland	3 (4)
Norway	3 (4)
Denmark	2 (3)
Romania	2 (3)
Sweden	1 (1)
Canada	1 (1)
Estonia	1 (1)
Ireland	1 (1)
Israel	1 (1)
Latvia	1 (1)
Luxembourg	1 (1)
Poland	1 (1)
South Africa	1 (1)
United Kingdom	1 (1)
Initiated (calendar year)	
2017	1 (1)
2019	8 (12)
2020	2 (3)
2021	2 (3)
2022	3 (4)
2023	34 (51)
2024	13 (19)
Start date not indicated	4 (6)
Disease type	
Cancer	32 (48)
Neurological and psychiatric disorders	10 (15)
Cardiovascular diseases	9 (13)
Metabolic diseases	6 (9)
Other NCDs	10 (16)
Prevention level	
Primary	18 (21)
Secondary	35 (41)
Tertiary	33 (38)

The majority of projects started in 2023 (51% of the total) and in 2024 (19%). For projects initiated from 2017 to 2023 and still ongoing, each year shows a maximum of 3 projects, except for 2019 which has 8 projects (12%).

In terms of targeted diseases, 48% of the projects address cancer, 15% focus on neurological and psychiatric diseases, 13% on cardiovascular diseases, 9% on metabolic disorders, and 16% on other NCDs, including both less represented disease areas and projects addressing NCDs in general, without a specific disease focus.

**Figure 2:**
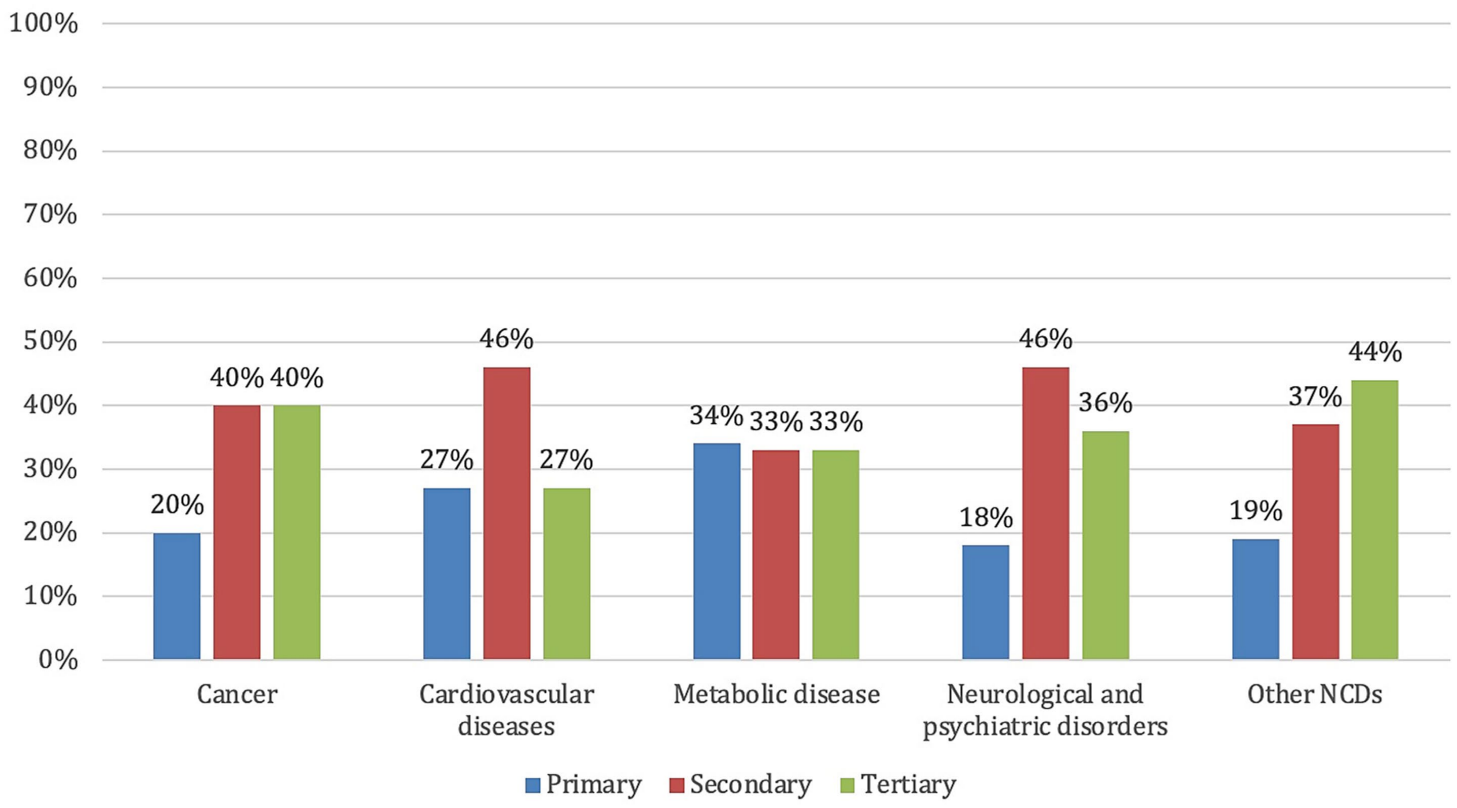
Distribution of the EU-funded projects on personalized prevention by disease type and prevention level.

Concerning the prevention levels, 21% of the projects focus on primary prevention, 41% on secondary prevention, and 38% on tertiary prevention. Figure 2 illustrates how prevention levels are distributed within each disease category, highlighting differences in the preventive approach adopted across disease types.

Regarding the main objectives of the identified research projects, 63% aimed to demonstrate the clinical validity or efficacy of specific biomarkers and stratification tools, followed by 13% focused on treatment personalization and 12% on screening and early diagnosis. Only 5% addressed implementation or participatory approaches, 3% focused on data sharing and infrastructure, and just 2% tackled ethical, legal, and social aspects, including issues related to equity and acceptability (Supplementary material S2).

### 3.2 Program funding

The mapping analysis reveals that research projects are funded by 3 main programs: Horizon Europe, which finances 51% of the projects, Horizon 2020 with 37%, and EU4Health with 12%. Table 2 provides a brief overview of these funding programs, along with detailed descriptive statistics for the projects they support.

**Table 2:** Distribution of research projects by funding programs and disease type.

Total projects	(*N* = 67)
Funding programs	*N* (%)
Horizon Europe	34 (51)
Cancer	15 (44)
Neurological and psychiatric disorders	5 (15)
Cardiovascular diseases	6 (18)
Metabolic diseases	4 (12)
Other NCDs	4 (12)
Horizon 2020	13 (19)
Cancer	5 (39)
Neurological and psychiatric disorders	3 (23)
Cardiovascular diseases	2 (15)
Metabolic diseases	0 (0)
Other NCDs	3 (23)
Horizon 2020 - ERA-NET Cofund	12 (18)
Cancer	6 (50)
Neurological and psychiatric disorders	2 (17)
Cardiovascular diseases	0 (0)
Metabolic diseases	2 (17)
Other NCDs	2 (17)
EU4Health	8 (12)
Cancer	6 (75)
Neurological and psychiatric disorders	0 (0)
Cardiovascular diseases	1 (13)
Metabolic diseases	0 (0)
Other NCDs	1 (13)

#### 3.2.1 Horizon Europe

Horizon Europe, the EU’s key funding program for research and innovation (operating from 2021 to 2027 with a total budget of €95.5 billion) (82), finances 34 (51%) of the mapped projects, with a total allocated funding of € 253,821,313.70 (Table 3). Specifically, 44% focus on cancer, 18% on cardiovascular diseases, 15% on neurological and psychiatric disorders, 12% on metabolic diseases, and 12% on other NCDs (Table 2).

**Table 3:** Detailed breakdown of European Commission funds allocated to personalized prevention projects.

Funding programs	Funds
Horizon Europe	€ 253,821,313.70
Cancer	€ 126,030,866.30
Neurological and psychiatric disorders	€ 41,049,919.37
Cardiovascular diseases	€ 26,133,149.81
Metabolic diseases	€ 26,540,068.25
Other NCDs	€ 34,067,310.00
Horizon 2020	€ 116,513,963.90
Cancer	€ 35,738,609.05
Neurological and psychiatric disorders	€ 26,734,936.84
Cardiovascular diseases	€ 26,929,723.26
Metabolic diseases	€ 0
Other NCDs	€ 27,110,694.79
EU4Health	€ 141,600,411.50
Cancer	€ 140,883,467.50
Neurological and psychiatric disorders	€ 0
Cardiovascular diseases	€ 524,444.00
Metabolic diseases	€ 0
Other NCDs	€ 192,500.00

#### 3.2.2 Horizon 2020

Horizon 2020, the EU’s funding program for research and innovation (operating from 2014 to 2020 with a total budget of €77 billion) (83), supports 13 (19%) of the mapped projects, with a total allocated funding of €116,513,963.90 (Table 3). Among these, 39% focus on cancer, 23% on neurological and psychiatric disorders, 15% on cardiovascular diseases, and 23% on other NCDs. Notably, no funding was allocated to metabolic diseases (Table 2).

In addition, Horizon 2020 - ERA-NET Cofund, a subprogram of Horizon 2020 involving Europe and beyond (84) funds 12 projects (18%), although no specific funding data are available for this program (Table 3). The projects supported by this subprogram are distributed as follows: 50% focus on cancer, 17% on neurological and psychiatric disorders, 17% on metabolic diseases, and 17% on other NCDs, while no projects target cardiovascular diseases (Table 2).

#### 3.2.3 Eu4Health

EU4Health, the EU’s health program designed to support health-related initiatives across the Union (operating from 2021 to 2027 with a total budget of €5.1 billion) (85), finances 8 (12%) of the mapped projects, with a total allocated funding of €141,600,411.50 (Table 3). Of these, 75% focus on cancer, 13% on cardiovascular diseases, and 13% on other NCDs. No project addresses neurological and psychiatric disorders or metabolic diseases (Table 2).

## 4 Discussion

**Figure 3:**
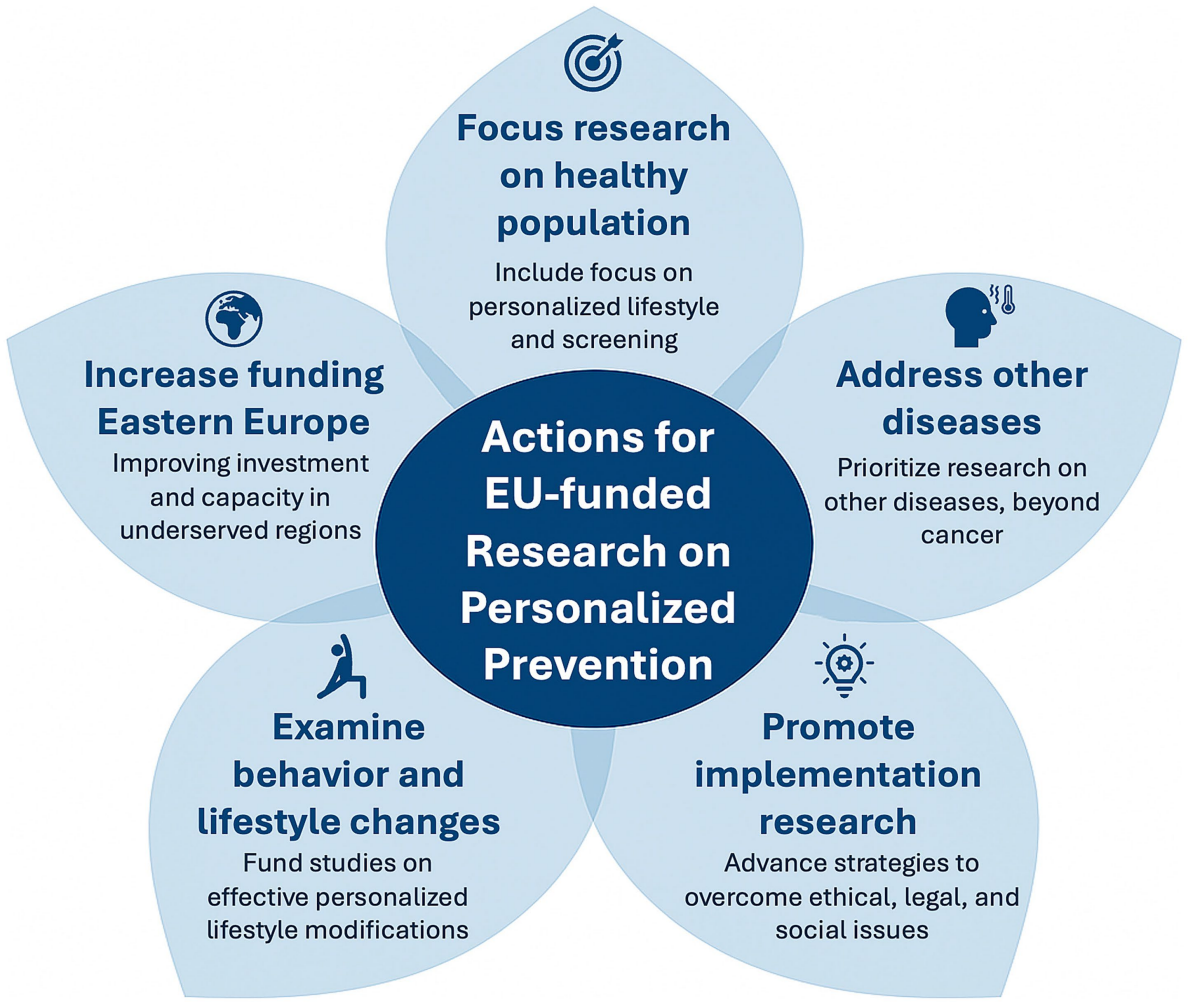
Recommended strategic actions for future EU-funded research on personalized prevention.

Our report highlights significant EU investment in research on personalized prevention, with the Horizon Europe program emerging as a central funding source. We identified 67 projects on personalized prevention funded by the European Commission, totaling approximately €511.9 million. These projects show a wide variety of objectives, thematic focuses, and methodological approaches. This underscores the EU’s commitment to addressing major health challenges through innovation in the field, while also revealing persistent gaps that require targeted efforts to overcome. Indeed, starting from the results of our review, we propose some general actions to be implemented for EU-funded research on personalized prevention, as summarized in Figure 3.

Horizon Europe, building on the foundations laid by Horizon 2020 (86) stands as a flagship initiative for research and development, positioning personalized prevention as a central component of its public health goals. This focus aligns with other EU-wide initiatives, such as the Cancer Mission (87) and the European Beating Cancer Plan (88), which both explicitly emphasize the role of personalized prevention in addressing Europe’s cancer burden. The prominence of cancer as a research focus within our mapped projects underscores this alignment, with personalized prevention highlighted in key policy documents. Notably, the Mission Board on Cancer’s report, “Conquering Cancer: Mission Possible” (89), prioritizes personalized prevention in its recommendations. Recommendation 2 proposes developing an EU-wide research program for polygenic risk scores, recommendation 4 suggests optimizing existing and developing new screening programs, and recommendation 5 advocates advancing personalized medicine for all cancer patients across Europe. Similarly, the “Europe’s Beating Cancer Plan” (90) identifies personalized prevention in Action 31.2, reinforcing the policy’s prioritization of this approach. In this context, the PROPHET project (7) will develop a roadmap for personalized prevention, while ECAC5 (28) will focus on updating the 5th edition of the European Code against Cancer (ECAC) based on the latest scientific evidence and expanding prevention measures to target diverse audiences. These advancements are built upon the foundation established by earlier initiatives, particularly ICPerMed (International Consortium for Personalized Medicine), started in 2016, which played a pioneering role in fostering collaboration and raising awareness of personalized medicine by bringing together over 40 international partners to support research, policy development, and the integration of personalized approaches into healthcare systems (9) Building on ICPerMed’s achievements, ERA PerMed (European Research Area for Personalized Medicine), active from 2017 to 2023, further strengthened European cooperation by funding over 110 interdisciplinary projects and enhancing collaboration among countries (91, 92) These efforts culminated in the launch of EP PerMed (European Partnership for Personalized Medicine) in 2023, which now consolidates and expands the progress made by the previous initiatives (58, 93).

However, despite these advancements, our findings indicate that conditions other than cancer have received comparatively less attention in the current landscape of personalized prevention EC-funded research. This trend is consistent with the relative lack of disease-specific European initiatives in neurological, psychiatric, cardiovascular or metabolic diseases, and also with trends observed in the scientific literature, including published primary studies and existing guidelines on the application of personalized prevention in these fields (94–96). This may be partly explained by the fact that prevention in these disease areas, even not personalized, remains a challenging and uncertain domain: although the molecular mechanisms underlying their pathogenesis are increasingly well understood, they are often influenced by lifestyle-related factors, which remain difficult to modify in a sustainable way to effectively reduce disease incidence (97–99) In contrast, cancer is characterized by a better-defined understanding of genetic predisposition, and by the existence of prevention programs, particularly in the domain of secondary prevention, such as organized screening programs, that are more firmly established and widely adopted across European healthcare systems.

Given the well-recognized burden, both globally and within Europe, of neurodegenerative and psychiatric disorders, which are steadily increasing in prevalence, as well as of cardiovascular and metabolic diseases, EU and EC priorities should include increased investment in research specifically targeting personalized prevention in these areas. This should encompass not only omics-based and biomedical approaches, but also primary studies aimed at evaluating the effectiveness of prevention strategies in real-world settings. Furthermore, there is a need for research on social and behavioral interventions that can effectively promote and sustain healthy lifestyles across diverse population groups and life stages. Further studies should also focus on the development and validation of tailored prevention programs and care pathways for individuals at increased risk of these diseases, by integrating individual predisposition with environmental and contextual factors. Dedicated funding mechanisms are essential to support such multidisciplinary research efforts and to generate actionable evidence to inform future public health strategies.

Our analysis of prevention levels within the 67 mapped projects reveals a marked emphasis on primary and secondary personalized prevention, when considered in total. Primary prevention strategies in personalized medicine, such as omics-based profiling, hold potential to identify high-risk individuals before disease onset, allowing for tailored lifestyle interventions (100, 101) Similarly, secondary prevention can leverage genomic screening to manage carriers of high-risk mutations, such as *BRCA*1/2 for breast cancer, thus enabling targeted early intervention (102) The focus on primary and secondary personalized prevention aligns with the substantial gap identified in the literature for personalized approaches targeting NCDs (96) and is evident in various key projects. For example, ONCODIR (54) addresses colorectal cancer through a multidisciplinary approach, integrating AI, health policy, social science, and omics-based research to develop personalized primary prevention programs across the EU. The LIVERAIM (73) project is creating an AI-based biomarker screening platform for the early diagnosis of liver fibrosis. MONALISA (70) uses liquid biopsies for managing and monitoring high-risk neuroblastoma in children, establishing new standards for sensitive and frequent monitoring. DEFINITIVE (61) validates HER2DX-guided treatment for early HER2-positive breast cancer, integrating these tools into clinical guidelines to promote the use of non-invasive diagnostic tools. Finally, MICROB-PREDICT (18) identifies microbiome-based biomarkers for personalized prediction of liver disease, integrating multi-omics data and conducting randomized clinical trials to validate these approaches.

By concentrating efforts on these areas, the EC is fostering evidence generation to support omics-based approaches that increase the precision of interventions, screenings, and diagnostics, which could ultimately reduce disease incidence and associated healthcare costs.

The significant investments in primary and secondary prevention underscore the EU’s commitment to generating robust clinical evidence to support omics-based approaches. These efforts are particularly focused on addressing the need for large-scale prospective studies to validate the clinical utility of personalized primary and secondary prevention, with specific attention to the integration of multi-omics data, longitudinal research on biomarker efficacy, and cost-effectiveness assessments of these personalized interventions (103–105). These efforts are vital to developing actionable, evidence-backed strategies that can inform public health policy and enhance healthcare outcomes.

In contrast, our findings indicate a lower investment in tertiary prevention research, which leverages omics sciences to optimize treatment for advanced NCDs. Pharmacogenomics, which tailors drug therapy to genetic profiles, stands as a leading example of personalized tertiary prevention and is already established in clinical settings through resources such as the Pharmacogenomic Knowledgebase (PharmGKB) and Clinical Pharmacogenetics Implementation Consortium (CPIC) guidelines (106). A clear example is the widespread implementation of *DPYD* genotyping to guide fluoropyrimidine therapy in colorectal cancer patients, allowing dose adjustments or alternative treatments for those with gene variants that increase the risk of severe toxicity (107–109). The lower priority given to tertiary prevention in EU-funded projects may reflect the advanced stage of pharmacogenomics implementation (110), suggesting a strategic shift by the EC toward areas with a greater need for clinical evidence, particularly primary and secondary preventive interventions. A possible explanation for the earlier and broader implementation of personalized approaches in the therapeutic domain lies in the relative simplicity of designing clinical trials for tertiary prevention, compared to primary and secondary prevention (111) Clinical trials in patients with established disease are generally easier to design and conduct. These individuals are often more willing to undergo genetic testing and experimental treatments, given their direct interest in improving clinical outcomes (112). This facilitates recruitment, adherence, and the measurement of endpoints such as treatment response or disease progression within shorter timeframes. Furthermore, the therapeutic domain has traditionally attracted significantly higher levels of investment, particularly from the pharmaceutical industry, which has a strong interest in developing and commercializing targeted treatments (113, 114).

Despite growing institutional attention toward personalized prevention, its large-scale implementation remains limited by persistent barriers. Key obstacles include ethical and legal concerns around data privacy, consent models, and potential discrimination; regulatory uncertainty across member states; and limited societal readiness to embrace stratified prevention (4, 115–118). The lack of shared governance models and public engagement strategies further complicates translation into practice.

Against this backdrop, our review of EU-funded research projects reveals that many of these critical dimensions are not yet systematically addressed. While several initiatives focus on biomarker discovery, risk prediction, and early detection, relatively few incorporate broader implementation-related aspects. For instance, only a minority of projects explicitly include social determinants of health, participatory approaches, or health economic evaluation in their design. Similarly, ethical, legal, and social issues (ELSI) are seldom integrated as core research components. This narrow scope may limit the future scalability and societal acceptability of proposed innovations. Furthermore, our analysis revealed important geographical patterns. Coordinating institutions were disproportionately located in Western Europe, with countries like Italy, the Netherlands, Germany, and France leading the majority of funded projects. In contrast, Eastern and Southern European countries were underrepresented, raising concerns about structural disparities in research capacity and funding access.

These findings suggest a disconnect between the complexity of real-world implementation and the current research agenda. Although the field of personalized prevention is expanding, it risks perpetuating existing gaps if future projects continue to focus predominantly on technical innovation without adequately considering the contextual, societal, and structural factors that shape its feasibility and impact.

While our study did not apply foresight methodologies, the insights derived from this review could serve as a foundation for future horizon scanning efforts aimed at anticipating shifts in research priorities and funding allocations, particularly in the context of upcoming EU research programs and the evolving roadmap of EP PerMed (58, 93).

One limitation of this study is that, although most of the identified projects will take months or years to conclude, the increasing annual funding for personalized prevention research may rapidly alter the landscape presented here. To address this, an iterative approach with annual reviews could be useful to capture such changes in a timely manner. Another limitation is the exclusive use of gray literature sources, which, while appropriate for identifying ongoing or recently funded projects, may have excluded relevant information from peer-reviewed publications. Additionally, the search was conducted only in English, potentially omitting projects described in other languages. Regarding data accuracy, all information was verified through official websites, but we cannot entirely exclude the possibility that unclear project objectives or the interdisciplinary nature of some projects may have influenced their categorization, despite the rigorous project selection and data collection carried out in a double-blind manner by the researchers. Furthermore, limitations in the CORDIS database suggest the presence of significant gaps, such as incomplete or outdated fields, which complicate project traceability (14). To mitigate these issues, we consulted experts in the field. Although their input was intended solely to complement the web search, it is important to note that these experts are part of the PROPHET consortium, which may have introduced a potential bias. Although we have attempted to reduce this risk by adopting a transparent and documented approach, this consideration should be taken into account when interpreting the results. However, the PROPHET consortium is intrinsically characterized by a high degree of geographical and professional diversity, which helps mitigate this risk. Finally, it is important to note that this overview did not assess the quality of the retrieved projects or their success in meeting the objectives, as this analysis was beyond the scope of our study.

In conclusion, our analysis underscores the EC’s significant investment in advancing personalized prevention, with a strong emphasis on primary and secondary prevention. This investment reflects a strategic priority for omics-based research to improve risk stratification, early diagnosis, and personalized preventive strategies, particularly in cancer. While the totality of primary and secondary prevention receives considerable attention, research on tertiary prevention is less prominent, likely due to its established clinical applications.

Despite this promising landscape, maximizing impact will require funding and project priorities to address critical gaps in the literature, especially in generating clinical utility evidence. Closing these gaps is essential for translating research into practice, ensuring that personalized prevention approaches are both evidence-backed and effectively integrated into EU healthcare systems. Indeed, this overview can inform stakeholders across the research and policy spectrum, guiding future funding calls, supporting policy alignment under EU missions, and enabling strategic coordination among research consortia.
