# Supplementary material for: Scanning the horizon of personalized prevention research: an overview of ongoing European funded initiatives
Source: Front Public Health. 2025 Aug 12;13:1561328. doi: 10.3389/fpubh.2025.1561328 (PMC12378169; doi:10.3389/fpubh.2025.1561328)
Supplement: Supplementary file 1 [file Data_Sheet_1.docx]

**Supplementary Material**

**Supplementary Table 1.** Overview of the ongoing EU-funded projects on personalized prevention.

| **Reference** | **Title (acronym)** | **Grant agreement ID** | **Project start date** | **Project end**  **date** | **Website** | **Coordinator country** | **Coordinating center** | **Coordinator activity type** | **Program** | **Total cost**  **(€)** | **EU contribution (€)** |
| --- | --- | --- | --- | --- | --- | --- | --- | --- | --- | --- | --- |
| (12) | PREvention of STroke in Intracerebral hemorrhaGE survivors with Atrial Fibrillation (PRESTIGE-AF) | 754517 | 01/12/17 | 30/11/24 | <https://www.imperial.ac.uk/brain-sciences/research/neurology/prestige-af/> | United Kingdom | Imperial College Of Science Technology And Medicine | Higher or secondary education establishment | H2020 | 6.958.896,00 | 6.958.896,00 |
| (13) | Genome, Environment, Microbiome & Metabolome in Autism: an integrated multi-omics systems biology approach to identify biomarkers for personalized treatment and primary prevention of Autism Spectr (GEMMA) | 825033 | 01/01/19 | 31/12/24 | <https://www.gemma-project.eu> | Italy | Fondazione Ebris | Research organization | H2020 | 14.225.757,50 | 14.225.757,50 |
| (14) | ERA-NET to support the Joint Programming in Neurodegenerative Diseases strategic plan (JPCOFUND2) | 825664 | 01/01/19 | 31/12/24 | <https://neurodegenerationresearch.eu> | France | Institut National De La Sante Et De La Recherche Medicale | Research organization | H2020 | 33.438.899,00 | 11.010.911,34 |
| (15) | MICROBiome-based biomarkers to PREDICT decompensation of liver cirrhosis and treatment response (MICROB-PREDICT) | 825694 | 01/01/19 | 31/03/25 | <https://microb-predict.eu> | Spain | European Foundation For The Study Of Chronic Liver Failure | Research organization | H2020 | 15.000.002,50 | 15.000.000,00 |
| (16) | Gut OncoMicrobiome Signatures (GOMS) associated with cancer incidence, prognosis and prediction of treatment response (ONCOBIOME) | 825410 | 01/01/19 | 30/06/24 | <https://www.oncobiome.eu> | France | Institut Gustave Roussy | Research organization | H2020 | 15.041.511,30 | 14.994.551,25 |
| (17) | The Brain Health Toolbox: Facilitating personalized decision-making for effective dementia prevention (Brain Health Toolbox) | 804371 | 01/02/19 | 31/07/24 | N.d. | Finland | Ita-Suomen Yliopisto | Higher or secondary education establishment | H2020 | 1.498.268,00 | 1.498.268,00 |
| (18) | Integrated iMMUnoprofiling of large adaptive CANcer patient cohorts (IMMUcan) | 821558 | 01/03/19 | 31/08/25 | <https://immucan.eu> | Belgium | European Organization For Research And Treatment Of Cancer Aisbl | Research organization | H2020 | 13.477.631,80 | 6.877.631,00 |
| (19) | Specific Imaging of Immune Cell Dynamics Using Novel Tracer Strategies (Immune-Image) | 831514 | 01/10/19 | 31/03/26 | <https://www.immune-image.eu> | Netherlands | Stichting Amsterdam Umc | Research organization | H2020 | 4.358.722,50 | 3.993.722,00 |
| (20) | Establishment and Exploitation of a European-Latin American Research Consortium towards Eradication of Preventable Gallbladder Cancer (EULAT Eradicate GBC) | 825741 | 01/12/19 | 30/11/25 | <https://www.klinikum.uni-heidelberg.de/eulat-eradicate-gbc> | Germany | Universitatsklinikum Heidelberg | Higher or secondary education establishment | H2020 | 4.675.932,30 | 4.675.932,30 |
| (21) | Personalized Prevention for Coronary Heart Disease (CoroPrevention) | 848056 | 01/01/20 | 31/12/26 | <https://coroprevention.eu> | Finland | Tampereen Korkeakoulusaatio Sr | Higher or secondary education establishment | H2020 | 22.641.162,00 | 19.970.827,26 |
| (22) | Monitoring multidimensional aspects of QUAlity of Life after cancer ImmunoTherapy -  an Open smart digital Platform for personalized prevention and patient management (QUALITOP) | 875171 | 01/01/20 | 30/06/24 | <https://h2020qualitop.liris.cnrs.fr/wordpress/index.php/project/> | France | Hospices Civils De Lyon | Public body (excluding research organizations and secondary or higher education establishments) | H2020 | 5.196.772,50 | 5.196.772,50 |
| (23) | Finding Endometriosis using Machine Learning (FEMaLe) | 101017562 | 01/01/21 | 31/12/24 | <https://findingendometriosis.eu> | Denmark | Aarhus Universitet | Higher or secondary education establishment | H2020 | 1.675.754,79 | 1.675.754,79 |
| (24) | International consortium for integrative genomics prediction (INTERVENE) | 101016775 | 01/01/21 | 31/12/25 | <https://www.interveneproject.eu> | Finland | Helsingin Yliopisto | Higher or secondary education establishment | H2020 | 10.434.940,00 | 10.434.940,00 |
| (25) | Revision and update of the European Code against Cancer (ECAC5) | 101075240 | 01/07/22 | 30/06/26 | N.d. | France | Centre International De Recherche Sur Le Cancer | Research organization | EU4H | 2.500.000,00 | 1.500.000,00 |
| (26) | A PeRsOnalized Prevention roadmap for the future HEalThcare (PROPHET) | 101057721 | 01/09/22 | 31/08/26 | <https://prophetproject.eu> | Italy | Università Cattolica del Sacro Cuore | Higher or secondary education establishment | HE | 413.540,00 | 378.415,00 |
| (27) | Building the EU Cancer and Public Health Genomics platform (CAN.HEAL) | 101080009 | 01/11/22 | 30/04/25 | <https://canheal.eu> | Belgium | Sciensano | Research organization | EU4H | 7.476.270,23 | 5.981.016,19 |
| (28) | Early dynamic screening for colorectal cancer via novel protein biomarkers reflecting biological initiation mechanisms (DIOPTRA) | 101096649 | 01/01/23 | 31/12/26 | N.d. | Greece | Erevnitiko Panepistimiako Institouto Systimaton Epikoinonion Kai Ypologiston | Research organization | HE | 13.647.600,80 | 13.646.550,50 |
| (29) | Epigenetic-genetic-mental health cascade based personalized prevention of non-communicable disease in adolescents diagnosed with autism (ETHEREAL) | 101095568 | 01/01/23 | 31/12/25 | N.d. | Ireland | University College Cork - National University Of Ireland | Higher or secondary education establishment | HE | 1.780.806,74 | 1.780.806,74 |
| (30) | Early Interception of Inflammatory-mediated Type 2 Diabetes (INTERCEPT-T2D) | 101095433 | 01/01/23 | 31/12/27 | N.d. | France | Institut National De La Sante Et De La Recherche Medicale | Research organization | HE | 8.090.972,75 | 8.090.972,75 |
| (31) | A European “shield” against colorectal cancer based on novel, more precise and affordable risk-based screening methods and viable policy pathways (ONCOSCREEN) | 101097036 | 01/01/23 | 31/12/26 | N.d. | Greece | Exus Software Monoprosopi Etairia Periorismenis Evthinis | Private for-profit entity (excluding higher or secondary education establishments) | HE | 12.972.076,30 | 12.972.076,00 |
| (32) | PANcreatic CAncer Initial Detection via liquid biopsy (PANCAID) | 101096309 | 01/01/23 | 31/12/27 | N.d. | Germany | Universitaetsklinikum Hamburg-Eppendorf | Higher or secondary education establishment | HE | 9.845.090,25 | 9.838.840,00 |
| (33) | Personalized Cancer Medicine for all EU citizens (PCM4EU) | 101079984 | 01/01/23 | 31/12/24 | N.d. | Netherlands | Academisch Ziekenhuis Leiden | Higher or secondary education establishment | EU4H | 3.679.327,26 | 2.943.458,99 |
| (34) | Early detection and screening of hematological malignancies (SANGUINE) | 101097026 | 01/01/23 | 31/12/25 | N.d. | Israel | Tel Aviv University | Higher or secondary education establishment | HE | 8.478.000,00 | 8.478.000,00 |
| (35) | Medical comorbidities in bipolar disorder: clinical validation of risk factors and biomarkers to improve prevention and treatment (BIPCOM) | ERAPERMED2022-087 | 01/02/23 | 31/01/26 | N.d. | Italy | IRCCS Istituto Centro San Giovanni Di Dio Fatebenefratelli Brescia | Private non-profit research organization | H2020 - ERA-NET Cofund | N.d. | N.d. |
| (36) | Prodromal DEtErminants for PhENoconversion of idiopathic RBD to alpha-synucleinopathies (PD, DLB and MSA) (DEEPEN-iRBD) | ERAPERMED2022-212 | 01/02/23 | 31/01/26 | N.d. | Italy | Università Degli Studi Di Milano | Higher or secondary education establishment | H2020 - ERA-NET Cofund | N.d. | N.d. |
| (37) | N6 - methyladenosine RNA modification in acute coronary syndrome (MAACS) | 101064175 | 01/03/23 | 28/02/25 | N.d. | Luxembourg | Luxembourg Institute Of Health | Research organization | HE | N.d. | 191.760,00 |
| (38) | Integration of heterogeneous Data and Evidence towards Regulatory and HTA Acceptance (IDERHA) | 101112135 | 01/04/23 | 31/03/28 | N.d. | Germany | Fraunhofer Gesellschaft Zur Forderung Der Angewandten Forschung Ev | Research organization | HE | 36.181.150,90 | 23.032.209,61 |
| (39) | Cancer multi-omics avatars for integrated precision medicine (LANTERN) | ERAPERMED2022-116 | 01/04/23 | 31/03/26 | N.d. | Italy | Fondazione Policlinico Universitario “A. Gemelli” IRCCS | Public organization | H2020 - ERA-NET Cofund | N.d. | N.d. |
| (40) | Personalizing the clinical decision making in ovarian cancer through patient-derived in vitro models (OVA-PDM) | ERAPERMED2022-141 | 01/04/23 | 31/03/26 | N.d. | Italy | Istituto Europeo di Oncologia s.r.l. | Private non-profit research organization | H2020 - ERA-NET Cofund | N.d. | N.d. |
| (41) | PRostate cancer Awareness and Initiative for Screening in the European Union (PRAISE-U) | 101101217 | 01/04/23 | 31/03/26 | N.d. | Netherlands | Stichting European Urological Foundation | Small or medium-size enterprise | EU4H | 12.311.328,10 | 9.849.062,50 |
| (42) | Personalized CANcer Primary Prevention research through Citizen Participation and digitally enabled social innovation (4P-CAN) | 101104432 | 01/05/23 | 30/04/27 | N.d. | Romania | Asociatia Centrul Pentru Inovatie In Medicina | Research organization | HE | 5.199.480,00 | 5.199.479,25 |
| (43) | Preventing lifetime obesity by early risk-factor identification, prognosis and intervention (eprObes) | 101080219 | 01/05/23 | 30/04/28 | N.d. | Spain | Consorcio Centro De Investigacion Biomedica En Red M.P. | Research organization | HE | 2.718.249,50 | 1.659.235,75 |
| (44) | Improving personalized glioblastoma care by intertwined immunomics and artificial intelligence approaches (IPerGlio) | ERAPERMED2022-245 | 01/05/23 | 30/04/26 | N.d. | Norway | Oslo University Hospital | Higher or secondary education establishment | H2020 - ERA-NET Cofund | N.d. | N.d. |
| (45) | Omics Approach for Personalized Prevention of Type 2 Diabetes Mellitus for African and European Populations (OPTIMA) | ERAPERMED2022-209) | 01/05/23 | 30/04/26 | N.d. | South Africa | Wits Health Consortium (Pty) Ltd | Public organization | H2020 - ERA-NET Cofund | N.d. | N.d. |
| (46) | Deciphering female’s SEx hormones - MIcrobiota interactions during a menstrual CYCLE for an efficient personalized medicine in cardiometabolic disorders (SEMICYCLE) | 101075624 | 01/05/23 | 30/04/28 | N.d. | Italy | Consiglio Nazionale Delle Ricerche | Research organization | HE | 1.499.286,00 | 1.499.286,00 |
| (47) | Body fluid proteome SIGnatures for persoNALized intervention to prevent cardiovascular and renal complications in diabetes (SIGNAL) | ERAPERMED2022-248 | 01/05/23 | 30/04/26 | N.d. | Denmark | Steno Diabetes Center Copenhagen (part of Region Hovedstaden) | Public organization | H2020 - ERA-NET Cofund | N.d. | N.d. |
| (48) | Urine biomarkers for bladder cancer diagnosis and surveillance: a multicentric study to assess the diagnostic accuracy of a comprehensive diagnostic tool (UBIOBCA) | ERAPERMED2022-096 | 01/05/23 | 30/04/26 | N.d. | Canada | Université de Sherbrooke | Higher or secondary education establishment | H2020 - ERA-NET Cofund | N.d. | N.d. |
| (49) | Developing trustworthy artificial intelligence (AI)-driven tools to predict vascular disease risk and progression (VASCUL-AID) | 101080947 | 01/05/23 | 30/04/29 | N.d. | Netherlands | Stichting VUMC | Research organization | HE | 5.969.125,00 | 5.969.125,00 |
| (50) | An integrated multi-omics signature of kidney fibrosis for CKD precision medicine (KidneySign) | ERAPERMED2022-202 | 02/05/23 | 01/05/26 | N.d. | France | RD Néphrologie SAS | Private -sme | H2020 - ERA-NET Cofund | N.d. | N.d. |
| (51) | Evidence-based Participatory Decision Making for Cancer Prevention through implementation research (ONCODIR) | 101104777 | 01/06/23 | 30/11/26 | N.d. | Greece | Ethniko Kentro Erevnas Kai Technologikis Anaptyxis | Research organization | HE | 7.893.636,25 | 7.893.636,25 |
| (52) | Centre for data enriched medicine (TEAMPERMED) | 101060011 | 01/09/23 | 31/08/29 | N.d. | Estonia | Tartu Ulikool | Higher or secondary education establishment | HE | 15.000.000,00 | 15.000.000,00 |
| (53) | Individualized care from early risk of cardiovascular disease to established heart failure (iCARE4CARDIOVASCULAR DISEASES) | 101112022 | 01/10/23 | 31/03/28 | N.d. | Netherlands | Universiteit Maastricht | Higher or secondary education establishment | HE | 20.323.025,40 | 9.942.458,81 |
| (54) | European action for the Diagnosis of Early Non-clinical Type 1 diabetes For disease Interception (EDENT1FI) | 101132379 | 01/11/23 | 31/10/28 | N.d. | Belgium | Katholieke Universiteit Leuven | Higher or secondary education establishment | HE | 5.004.582,50 | 2.754.582,50 |
| (55) | European Partnership for Personalized Medicine (EP PerMed) | 101137129 | 01/11/23 | 31/10/33 | <https://www.eppermed.eu> | Germany | Deutsches Zentrum Fur Luft - Und Raumfahrt Ev | Research organization | HE | 54.230.375,00 | 16.269.112,50 |
| (56) | Prediction of Alzheimer’s disease using an ai driven screening platform (PREDICTOM) | 101132356 | 01/11/23 | 31/10/27 | N.d. | Norway | Helse Stavanger Hf | Other | HE | 18.103.112,80 | 8.449.326,38 |
| (57) | Prediction and prevention of cardiovascular diseases in pre- and type 2 diabetes (PROVIDE) | 101128983 | 01/11/23 | 31/10/26 | N.d. | Italy | Università Degli Studi dell'Aquila | Higher or secondary education establishment | EU4H | 655.560,11 | 524.444,00 |
| (58) | Diagnostic HER2DX-guided treatment for patients with early-stage HER2-positive breast cancer (DEFINITIVE) | 101136953 | 01/12/23 | 30/11/28 | N.d. | Spain | Fundacio De Recerca Clinic Barcelona-Institut D Investigacions Biomediques August Pi I Sunyer | Research organization | HE | 7.999.930,00 | 7.999.930,00 |
| (59) | GRIP on MASH: Global Research Initiative for Patient Screening on MASH (GRIPonMASH) | 101132946 | 01/12/23 | 30/11/27 | N.d. | Netherlands | Universitair Medisch Centrum Utrecht | Higher or secondary education establishment | HE | 25.913.513,30 | 14.035.277,25 |
| (60) | Targeting tumor-host interactions in pediatric malignant gliomas to reinvigorate immunity and improve radio- and immunotherapy efficacy (HIT-GLIO) | 101136835 | 01/12/23 | 30/11/27 | N.d. | Poland | Instytut Biologii Doswiadczalnej Im. M. Nenckiego Polskiej Akademii Nauk | Research organization | HE | 1.782.000,00 | 1.782.000,00 |
| (61) | Well-being improvement through the Integration of healthcare and reSearch Data and models with Out border for chronic iMmune-mediated diseases (WISDOM) | 101137154 | 01/12/23 | 30/11/28 | N.d. | Sweden | Karolinska Institutet | Higher or secondary education establishment | HE | 2.419.782,50 | 2.419.782,50 |
| (62) | Real-world implementation, deployment and validation of early detection tools and lifestyle enhancement (AD-RIDDLE) | 101132933 | 01/01/24 | 31/12/28 | N.d. | Sweden | Region Stockholm | Public body (excluding research organizations and secondary or higher education establishments) | HE | 29.051.202,5 | 12.090.250,00 |
| (63) | Prediction, Monitoring and Personalized Recommendations for Prevention and Relief of Dementia and Frailty (COMFORTAGE) | 101137301 | 01/01/24 | 31/12/27 | N.d. | Greece | University Of Piraeus Research Center | Higher or secondary education establishment | HE | 17.591.490,00 | 17.591.490,00 |
| (64) | A federated paradigm of real-world data sources utilization for the empowerment of diagnosis, prognosis and risk assessment of cardiovascular conditions (CARDIOVASCULAR DISEASESLINK) | 101137278 | 01/01/24 | 31/12/27 | N.d. | Romania | Software Imagination & Vision Srl | Private for-profit entity (excluding higher or secondary education establishments) | HE | 928.750,00 | 928.750,00 |
| (65) | Endoscopic brush cytology and single cell clinal dynamics of early esophageal adenocarcinoma for detecting cost effective surveillance strategies and prediction of cancer recurrence (ENDEAVOR) | 101136935 | 01/01/24 | 31/12/28 | N.d. | Belgium | Universiteit Antwerpen | Higher or secondary education establishment | HE | 6.726.488,69 | 6.726.488,69 |
| (66) | HetERogeneous sEmantic Data integratIon for the guT-bRain interplay (HEREDITARY) | 101137074 | 01/01/24 | 31/12/27 | N.d. | Italy | Università degli Studi di Padova | Higher or secondary education establishment | HE | 1.138.046,25 | 1.138.046,25 |
| (67) | A SIOPEN pragmatic clinical trial to MOnitor NeuroblastomA relapse with LIquid biopsy Sensitive Analysis (MONALISA) | 101137028 | 01/01/24 | 31/12/28 | N.d. | Belgium | Siop Europe | Other | HE | 7.232.386,25 | 7.232.386,25 |
| (68) | Next generation tools for genome-centric multimodal data integration in personalized cardiovascular medicine (NextGen) | 101136962 | 01/01/24 | 31/12/27 | N.d. | Netherlands | Universitair Medisch Centrum Utrecht | Higher or secondary education establishment | HE | 7.601.771,25 | 7.601.770,00 |
| (69) | BioMed Alliance EXpertise in health across legislation at EU level (EXHALE) | 101176496 | 01/02/24 | 31/12/24 | N.d. | Belgium | Biomedical Alliance In Europe | Other | EU4H | 320.985,73 | 192.500,00 |
| (70) | A Biomarker-Based Platform for Early Diagnosis of Chronic Liver Disease to Enable Personalized Therapy (LIVERAIM) | 101132901 | 01/03/24 | 28/02/30 | N.d. | Spain | Fundacio De Recerca Clinic Barcelona-Institut D Investigacions Biomediques August Pi I Sunyer | Research organization | HE | 24.789.953,90 | 13.854.592,75 |
| (71) | Residual disease assessment in hematologic malignancies to improve patient-relevant outcomes across Europe (RESOLVE) | 101136502 | 01/04/24 | 31/03/29 | N.d. | Germany | Medizinische Hochschule Hannover | Higher or secondary education establishment | HE | 2.188.010,00 | 2.188.010,00 |
| (72) | Implementation of cancer screening programs (EUCanScreen) | 101162959 | 01/06/24 | 31/05/28 | N.d. | Latvia | Latvijas Universitate | Higher or secondary education establishment | EU4H | 38.749.935,30 | 30.999.929,42 |
| (73) | SPatial Analysis of Cancer Evolution in the Tumor Immune MicroEnvironment (SPACETIME) | 101136552 | 01/08/24 | 31/07/29 | N.d. | Netherlands | Stichting Amsterdam UMC | Research organization | HE | 5.186.667,75 | 5.186.667,00 |
| (74) | The European Comprehensive Cancer Centre Network (EUnetCCC JA) | 101183407 | 01/10/24 | 30/09/28 | N.d. | France | Institut National Du Cancer Gip | Public organization | EU4H | 112.012.504,00 | 89.610.000,35 |
| (75) | Glioma invasion assays as a predictive tool for personalized glioma medicine (Glioma-PerMed) | ERAPERMED2022-214 | N.d. | N.d. | N.d. | Germany | University of Dusseldorf | Higher or secondary education establishment | H2020 - ERA-NET Cofund | N.d. | N.d. |
| (76) | miRNA as biomarkers in early detection and personalized treatment in ovarian cancer (miRPOC) | ERAPERMED2022-073) | N.d. | N.d. | N.d. | Norway | Oslo University Hospital | Higher or secondary education establishment | H2020 - ERA-NET Cofund | N.d. | N.d. |
| (77) | A multi-omics stratification and a non-invasive tool for early recognition of triple negative and Her2+ breast cancer patients responders to neoadjuvant therapy (PORTRAIT) | ERAPERMED2022-114 | N.d. | N.d. | N.d. | Italy | IRCCS Istituto Romagnolo per lo Studio dei Tumori "Dino Amadori" | Public organization | H2020 - ERA-NET Cofund | N.d. | N.d. |
| (78) | Tumor-host interactions in liver cancer of childhood and adults (THRIVE) | 101136622 | N.d. | N.d. | N.d. | Spain | Fundacio De Recerca Clinic Barcelona-Institut D Investigacions Biomediques August Pi I Sunyer | Research organization | HE | N.d. | N.d. |

N.d. = no data

**Supplementary Table 2.** Key characteristics of the ongoing EU-funded projects on personalized prevention.

| **Reference** | **Title (acronym)** | **Type of disease** | **Prevention level** | **General objective** | **Main topic** |
| --- | --- | --- | --- | --- | --- |
| (70) | A Biomarker-Based Platform for Early Diagnosis of Chronic Liver Disease to Enable Personalized Therapy (LIVERAIM) | Cancer | Secondary | To combat the rising burden of liver disease and cancer in Europe by: 1) developing and validating an AI-powered biomarker-based screening platform for early detection of liver fibrosis; 2) conducting analysis on biobank samples from 40,000 individuals and a randomized controlled trial (RCT) with 100,000 participants across six EU countries; 3) implementing personalized therapeutic interventions to halt fibrosis progression and improve health outcomes. | Biomarkers and risk stratification |
| (31) | A European “shield” against colorectal cancer based on novel, more precise and affordable risk-based screening methods and viable policy pathways (ONCOSCREEN) | Cancer | Secondary | To increase participation in colorectal cancer (CRC) screening by developing and validating: 1) a risk-based, population-level stratification methodology considering genetic, socioeconomic, and environmental factors; 2) accurate, non-invasive, and cost-effective screening technologies with high sensitivity and specificity; 3) AI-driven tools for early detection and personalized risk stratification; 4) a mobile app for self-monitoring and CRC awareness, alongside an Intelligent Analytics dashboard for policymakers. | Biomarkers and risk stratification |
| (64) | A federated paradigm of real-world data sources utilization for the empowerment of diagnosis, prognosis and risk assessment of cardiovascular conditions (CARDIOVASCULAR DISEASESLINK) | Cardiovascular diseases | Primary, secondary | To revolutionize cardiovascular disease (CVD) prevention and care by: 1) implementing a privacy-by-design European-wide federated platform for secure integration and sharing of clinical, genetic, and real-world data; 2) developing AI-powered precision medicine tools for diagnosis, risk stratification, and treatment of seven cardiovascular conditions across seven countries; 3) validating tools in five countries to demonstrate their impact on healthcare systems and citizens; 4) promoting widespread adoption through best practices, cost-effectiveness analysis, and awareness campaigns, aiming to reduce CVD burden and advance personalized care. | Biomarkers and risk stratification |
| (77) | A multi-omics stratification and a non-invasive tool for early recognition of triple negative and Her2+ breast cancer patients responders to neoadjuvant therapy (PORTRAIT) | Cancer | Primary, tertiary | To improve the personalization and effectiveness of neoadjuvant therapy (NAT) for Triple Negative (TN) and Her2+ breast cancer by: 1) developing a multidimensional patient stratification system integrating host and tumor microbiota, clinical data, and omics approaches (e.g., volatilomics, proteomics, NGS); 2) creating a non-invasive predictive tool for identifying responders vs. non-responders using innovative biomarkers and Systems Biology algorithms; 3) testing personalized therapeutic approaches on 3D models to explore interactions between microbiota and treatment outcomes; 4) ensuring ethical compliance with data protection (GDPR) and addressing ethico-legal aspects of organoid-based research. | Biomarkers and risk stratification |
| (26) | A PeRsOnalized Prevention roadmap for the future HEalThcare (PROPHET) | Other chronic diseases | Primary, secondary, tertiary | To support the implementation of personalized prevention programs for chronic diseases by: 1) developing a Strategic Research and Innovation Agenda (SRIA) to guide innovative, sustainable, and effective personalized prevention strategies; 2) mapping and evaluating advances in personalized prevention technologies, including omics and digital tools, in collaboration with stakeholders; 3) designing a holistic framework (PROPHET Framework) for assessing and adopting personalized prevention approaches in public health systems; 4) building capacity through guidelines, healthcare professional engagement, and health literacy initiatives to promote adoption and citizen understanding of personalized prevention benefits. | Implementation and participation |
| (67) | A SIOPEN pragmatic clinical trial to MOnitor NeuroblastomA relapse with LIquid biopsy Sensitive Analysis (MONALISA) | Cancer | Secondary, tertiary | To improve monitoring and treatment of high-risk neuroblastoma in children by: 1) establishing liquid biopsies as a less invasive, sensitive, and frequent monitoring standard compared to current imaging and bone marrow assessments; 2) detecting molecular progression or relapse early through tumor-derived DNA and RNA markers in blood; 3) validating the effectiveness of liquid biopsies in a pragmatic randomized clinical trial (RCT) to improve survival through earlier diagnosis and intervention; 4) developing a digital decision support tool for oncologists and assessing patient-reported outcomes to evaluate quality of life benefits; 5) advancing personalized medicine by integrating molecular diagnostics into treatment monitoring. | Screening and early diagnosis |
| (50) | An integrated multi-omics signature of kidney fibrosis for CKD precision medicine (KidneySign) | Other chronic diseases | Tertiary | To develop and validate an innovative multimodal protein-based signature using translational Big Data, integrating proteomics and peptidomics from various body fluids (urine, serum, plasma). This signature will accurately reflect kidney fibrosis and predict the risk of chronic kidney disease (CKD) progression. KidneySign aims to create a clinical decision support system for personalized nephroprotective therapies, enabling precision medicine. The project will utilize existing patient cohorts and biobanks along with a prospective clinical study to evaluate these markers. It will also address potential ethical and societal challenges related to their application within the ERA-PerMed network. | Biomarkers and risk stratification |
| (69) | BioMed Alliance EXpertise in health across legislation at EU level (EXHALE) | Other chronic diseases | Tertiary | The Biomedical Alliance in Europe (BioMed Alliance) aims to enhance the capacity and advocacy of its medical expert community by strengthening their involvement in EU decision-making processes. Through the EXHALE initiative, BioMed Alliance focuses on building capacity for medical specialists to effectively share their expertise with legislators. This involves supporting the implementation of EU health priorities such as health data sharing, Medical Devices Regulation, In Vitro Diagnostics Regulation, and Health Technology Assessment Regulation. EXHALE bridges the gap between clinical practice and EU-level decision-making by facilitating networking and cooperation among stakeholders. | Data infrastructure and sharing |
| (47) | Body fluid proteome SIGnatures for persoNALized intervention to prevent cardiovascular and renal complications in diabetes (SIGNAL) | Metabolic diseases | Tertiary | To evaluate and establish predictive biomarkers for guiding anti-diabetic treatment to prevent chronic kidney and cardiovascular disease. This involves compiling available resources, re-analyzing data to establish optimal biomarker profiles with predictive value, validating them independently, and assessing the health economics of clinical implementation. The availability of clinical resources, datasets from thousands of patients, and the expertise of consortium partners ensures the prompt advancement towards clinical implementation of these predictive biomarkers, enabling personalized treatment for diabetic patients. | Biomarkers and risk stratification |
| (27) | Building the EU Cancer and Public Health Genomics platform (CAN.HEAL) | Cancer | Primary, secondary, tertiary | To integrate genomics technologies into clinical and public health frameworks for improved cancer prevention, diagnosis, and treatment. This involves applying ‘next generation sequencing’ for genetic profiling, harmonizing data interpretation, developing polygenic risk scores, enhancing remote genetic counselling, and integrating the Genome of Europe biobanking initiative into public health genomics. The goal is to create an aligned, comprehensive approach to personalized medicine for cancer. | Biomarkers and risk stratification |
| (39) | Cancer multi-omics avatars for integrated precision medicine (LANTERN) | Cancer | Tertiary | To develop accurate predictive models for lung cancer patients using omics data, clinical factors, and advanced imaging features to create individualized patient avatars. These avatars will assist in clinical decision-making, enabling new paradigms of care and research by evaluating previously unexplored variable links. | Biomarkers and risk stratification |
| (52) | Centre for data enriched medicine (TEAMPERMED) | Other chronic diseases | N.d. | To become an internationally recognized leader in personalized medicine by using genomics and electronic health data, developing AI tools and protocols, and establishing a scalable framework for clinical guidelines and decision support systems. With guidance from leading experts and support from the EC and the Republic of Estonia, TeamPerMed will drive digital transformation in healthcare, improve individual patient outcomes, and enhance the quality of healthcare across Europe. | Implementation and participation |
| (46) | Deciphering female’s SEx hormones - MIcrobiota interactions during a menstrual CYCLE for an efficient personalized medicine in cardiometabolic disorders (SEMICYCLE) | Cardiovascular diseases | N.d. | To systematically investigate the interactions between female sex hormones and the microbiota to identify key microbiota regulators influencing glycemic and lipid metabolism in women. This research will track 300 healthy women over one menstrual cycle and generate comprehensive cross-omics data. The findings will provide new insights into CMD mechanisms in women, offering pathways for personalized prevention, diagnosis, and treatment of cardiometabolic disorders. | Screening and early diagnosis |
| (49) | Developing trustworthy artificial intelligence (AI)-driven tools to predict vascular disease risk and progression (VASCUL-AID) | Cardiovascular diseases | Primary, tertiary | To revolutionize the management of vascular diseases by predicting their progression through an artificial intelligence-based platform that can predict the risk and progression of vascular diseases such as Abdominal Aortic Aneurysm (AAA) and Peripheral Arterial Disease (PAD). | Biomarkers and risk stratification |
| (58) | Diagnostic HER2DX-guided treatment for patients with early-stage HER2-positive breast cancer (DEFINITIVE) | Cancer | Secondary | To prospectively validate the clinical effectiveness of the HER2DX test for personalizing treatment in early-stage HER2+ breast cancer, aiming to reduce toxicities, improve quality of life, and lower costs. A pragmatic clinical trial involving 304 patients in 44 centers in 7 European countries and associated countries will be conducted, randomizing participants to receive HER2DX-guided treatment versus standard care. The project aims to incorporate HER2DX testing into clinical guidelines and healthcare systems, promoting the use of noninvasive diagnostic tools in oncology and genomics. | Treatment personalization |
| (34) | Early detection and screening of hematological malignancies (SANGUINE) | Cancer | Secondary | To address hematologic malignancies by developing a minimally invasive blood test using epigenetic biomarkers. The test, based on a customized microarray (HemaChip), offers high sensitivity at low cost, ideal for large-scale screening. | Biomarkers and risk stratification |
| (28) | Early dynamic screening for colorectal cancer via novel protein biomarkers reflecting biological initiation mechanisms (DIOPTRA) | Cancer | Secondary | To develop a front-line screening tool using risk factors and protein biomarkers to identify individuals at high risk for colorectal cancer (CRC). The tool will analyze tissue and blood samples for prognostic proteins detectable through standard bloodwork, prompting further evaluation like a colonoscopy if necessary. AI will be used to enhance prognostic assessment, and personalized behavioral changes will be promoted based on modifiable risk factors. | Biomarkers and risk stratification |
| (30) | Early Interception of Inflammatory-mediated Type 2 Diabetes (INTERCEPT-T2D) | Metabolic diseases | Tertiary | To understand the role of inflammation in the onset and progression of type 2 diabetes (T2D) complications. By combining advanced genomics, clinical data and targeted therapies, the project aims to identify high-risk patients and develop personalized prevention strategies. A phase II study will test an anti-inflammatory therapy to prevent T2D complications. | Treatment personalization |
| (65) | Endoscopic brush cytology and single cell clinal dynamics of early esophageal adenocarcinoma for detecting cost effective surveillance strategies and prediction of cancer recurrence (ENDEAVOR) | Cancer | Secondary | To develop an innovative risk stratification method for early-stage esophageal adenocarcinoma (EAC) in Barrett’s Esophagus (BE) patients. Current endoscopic treatments result in over-treatment and under-treatment due to the inability to predict disease recurrence or progression. The project aims to use minimally invasive cell collection and single-cell genomics analysis to identify which patients are at higher risk of developing invasive cancer. | Biomarkers and risk stratification |
| (29) | Epigenetic-genetic-mental health cascade based personalized prevention of non-communicable disease in adolescents diagnosed with autism (ETHEREAL) | Neurological and psychiatric disorders | Tertiary | To address Mental Health Disorders (MHD) in adolescents with Autism Spectrum Disorders (ASD) by developing a personalized, flexible service model. Using a combination of epigenetic, genetic, and metabolomics data, the model empowers these adolescents to manage their environment and access community care. | Other |
| (14) | ERA-NET to support the Joint Programming in Neurodegenerative Diseases strategic plan (JPCOFUND2) | Neurological and psychiatric disorders | N.d. | Exploring personalized diagnosis, prevention, and treatment for neurodegenerative diseases. It also includes activities such as aligning national research strategies and strengthening research infrastructure. | Screening and early diagnosis |
| (20) | Establishment and Exploitation of a European-Latin American Research Consortium towards Eradication of Preventable Gallbladder Cancer (EULAT Eradicate GBC) | Cancer | Primary, secondary, tertiary | To improve the accuracy of GBC risk estimation and early detection by integrating geographic, environmental, lifestyle, and molecular factors. It will build a European–Latin American biorepository, identify novel biomarkers, develop a risk score, and explore targeted therapies. The resulting data will guide prevention strategies and influence national health policies. | Biomarkers and risk stratification |
| (54) | European action for the Diagnosis of Early Non-clinical Type 1 diabetes For disease Interception (EDENT1FI) | Metabolic diseases | Secondary | To establish a screening platform across Europe for early detection of Type 1 Diabetes (T1D) in children and adolescents. It aims to reduce clinical severity by identifying T1D at pre-clinical stages, using advanced tools and technologies. The project will also evaluate the impact of screening across diverse health systems and support the integration of early T1D diagnosis into regular healthcare. This effort will set new standards for T1D care and potentially benefit other chronic childhood diseases. | Screening and early diagnosis |
| (55) | European Partnership for Personalized Medicine (EP PerMed) | Other chronic diseases | Primary, secondary, tertiary | Is a European Partnership dedicated to Personalized Medicine (PM), aiming to facilitate and accelerate the implementation of PM into sustainable health systems. It supports demonstration projects, fosters patient involvement, and collaborates with medical societies, infrastructures, and international partners. The partnership aligns its activities with the Strategic Research and Innovation Agenda for PM (SRIA), developed with input from PM experts and the European Commission. EP PerMed also supports ERA PerMed and other projects to become successful innovations in healthcare. Through Joint Transnational Calls and other initiatives, EP PerMed serves as a global platform for scientific dialogue and strategic alignment, informing and engaging the public, patients, healthcare providers, and payers about the latest PM options. | Data infrastructure and sharing |
| (51) | Evidence-based Participatory Decision Making for Cancer Prevention through implementation research (ONCODIR) | Cancer | Primary | To address colorectal cancer (CRC) through a multidisciplinary approach, incorporating AI, health policy, social science, and omics-based research to develop evidence-based and personalized prevention programs. It focuses on ensuring cost effectiveness and affordability, testing these approaches across multiple EU countries | Ethical, legal, and social aspects |
| (23) | Finding Endometriosis using Machine Learning (FEMaLe) | Other chronic diseases | Secondary | To revitalize the 'P4 Medicine' framework by developing the Scalable Multi-Omics Platform (SMOP). This platform converts multi-omics data into personalized predictive models for managing endometriosis, facilitating shared decision-making between patients and healthcare providers. It includes tools like a mobile health app, clinical decision support tools for different healthcare providers, and a computer vision-based software for surgery. The project seeks to improve patient outcomes and reduce treatment costs by at least 20% for health maintenance organizations, with a vision for sustained impact beyond the project period through ethical and legal frameworks. | Biomarkers and risk stratification |
| (13) | Genome, Environment, Microbiome & Metabolome in Autism: an integrated multi-omics systems biology approach to identify biomarkers for personalized treatment and primary prevention of Autism Spectr (GEMMA) | Neurological and psychiatric disorders | Secondary, tertiary | To integrate a multi-omics approach with environmental data to explore the role of gut microbiota in the onset and progression of Autism Spectrum Disorders (ASD) in at-risk infants. By following 600 infants from birth and studying their microbiome, GEMMA seeks to provide mechanistic evidence linking abnormal gut microbiota with epigenetic changes affecting immune function. The project supports personalized treatment and disease interception strategies by modulating the microbiome to restore immune balance. Biomarkers identified could revolutionize ASD diagnosis and patient stratification for other conditions involving the interplay between genome, microbiome, and metabolism. The project will create a unique biobank of over 16,000 biospecimens for future research. | Biomarkers and risk stratification |
| (75) | Glioma invasion assays as a predictive tool for personalized glioma medicine (Glioma-PerMed) | Cancer | Tertiary | To address the challenges of Glioblastoma multiforme (GBM) by developing personalized glioma invasion assays using human brain organoids and zebrafish. This involves establishing rapid, efficient methods to predict glioma stem cell (GSC) invasion and correlate these behaviors with the genomics and transcriptomics profiles of individual patients. The project seeks to identify patient-specific therapeutic molecules that can target GSC invasions in clinical settings. This strategy aims to overcome the heterogeneity and aggressive behavior of GBM, ultimately leading to personalized treatments and improved outcomes for GBM patients. | Biomarkers and risk stratification |
| (59) | GRIP on MASH: Global Research Initiative for Patient Screening on MASH (GRIPonMASH) | Metabolic diseases | Secondary | It will develop a platform in primary care settings across Europe to screen at-risk patients, utilize AI decision support tools and multi-OMICs analysis for better patient stratification, and explore non-invasive diagnostic alternatives. The project aims to transform healthcare for Metabolic dysfunction-Associated Steatotic Liver Disease (MASLD), reduce the disease burden, and improve patient outcomes. | Biomarkers and risk stratification |
| (16) | Gut OncoMicrobiome Signatures (GOMS) associated with cancer incidence, prognosis and prediction of treatment response (ONCOBIOME) | Cancer | Primary, secondary, tertiary | To understand and characterize gut microbiome signatures (GOMS) associated with cancer across multiple cancer types, including breast, colorectal, melanoma, and lung cancers. By analyzing over 9,000 cancer patients, the project seeks to identify GOMS related to cancer occurrence, progression, and response to therapies. These findings will inform the development of predictive tools for cancer and guide the use of probiotics in cancer prevention. | Biomarkers and risk stratification |
| (66) | HetERogeneous sEmantic Data integratIon for the guT-bRain interplay (HEREDITARY) | Neurological and psychiatric disorders | Primary | To transform disease detection and treatment by integrating multimodal health data in a secure and privacy-compliant framework. It focuses on federated analytics to identify new risk factors and treatment responses for neurodegenerative and gut microbiome disorders. The project harmonizes clinical, genomics, and environmental data on a large scale, ensuring that patients and the public play a central role in guiding research. | Treatment personalization |
| (72) | Implementation of cancer screening programs (EUCanScreen) | Cancer | Secondary | To enhance cancer screening across Europe by implementing high-quality, evidence-based programs for breast, cervical, colorectal, lung, prostate, and gastric cancers. It focuses on ensuring proper governance, sustainability, and equal access for all eligible EU citizens, reducing cancer disparities. The project will improve data collection, monitoring, and capacity building in cancer screening. | Screening and early diagnosis |
| (44) | Improving personalized glioblastoma care by intertwined immunomics and artificial intelligence approaches (IPerGlio) | Cancer | Tertiary | To enhance the treatment and quality of life for glioblastoma (GBM) patients by integrating clinical, immunological, and lifestyle/environmental data using AI technologies to develop prognostic biomarkers. The goal is to guide personalized treatment decisions, particularly with immunotherapy, improving survival and disease management. | Biomarkers and risk stratification |
| (53) | Individualized care from early risk of cardiovascular disease to established heart failure (iCARE4CVD) | Cardiovascular diseases | Primary, secondary, tertiary | To tackle the growing burden of cardiovascular disease (CVD) by improving care pathways across all stages from early risk identification to established heart failure (HF). The project will achieve this through early diagnosis, risk stratification, and prediction of treatment response using a large dataset of biomarkers and artificial intelligence. | Biomarkers and risk stratification |
| (18) | Integrated iMMUnoprofiling of large adaptive CANcer patient cohorts (IMMUcan) | Cancer | Tertiary | To establish an integrated European immuno-oncology platform by collecting high-quality human biological material and clinical data from various cancer types and patients. The project will perform in-depth immunoprofiling and analyze data to identify predictive biomarkers for immunotherapy response or develop combination therapies. It will create a centralized biobank and IT platform, ensuring sustainability and accessibility for future research. | Biomarkers and risk stratification |
| (38) | Integration of heterogeneous Data and Evidence towards Regulatory and HTA Acceptance (IDERHA) | Cancer | Primary, secondary | With a focus on semantic interpretation standards, data quality, ethics, and transferability, the project implements use cases along the lung cancer patient pathway, demonstrating the added value of multi-modal data aggregation and analysis. Using clinical data, CT images, and digital biomarkers, the project enables remote patient monitoring and supports joint decision-making between patients and healthcare professionals, promoting personalized prognosis and improving public health outcomes. | Biomarkers and risk stratification |
| (24) | International consortium for integrative genomics prediction (INTERVENE) | Other chronic diseases | Primary, secondary, tertiary | To develop next-generation tools for disease prevention, diagnosis, and personalized treatment using a combined US-European genomics data pool. By integrating omics data into genetic risk scores, the project will provide unprecedented potential for complex and rare diseases. It will demonstrate the benefits of AI on integrative genetic scores (IGS) in key disease areas and develop a framework for their ethical and legal clinical integration. The IGS4EU platform will support IGS application through public-private partnerships, facilitating widespread adoption in clinical practice. | Biomarkers and risk stratification |
| (35) | Medical comorbidities in bipolar disorder: clinical validation of risk factors and biomarkers to improve prevention and treatment (BIPCOM) | Neurological and psychiatric disorders | Secondary | To use omics approaches to study comorbidities in bipolar disorder, analyzing data from Nordic biobanks and existing patient datasets. It develops a Clinical Support Tool for personalized clinical decision-making, enhancing prevention and treatment of BD. the project engages stakeholders to ensure the impact and transferability of its results. | Treatment personalization |
| (15) | MICROBiome-based biomarkers to PREDICT decompensation of liver cirrhosis and treatment response (MICROB-PREDICT) | Other chronic diseases | Secondary, tertiary | To improve understanding of the microbiome’s role in liver cirrhosis progression and acute-on-chronic liver failure (ACLF). The project focuses on identifying microbiome-based biomarkers for personalized prediction and response to treatment, developing new point-of-care tests and nano biosensors, and validating them in a randomized controlled trial (RCT). By integrating data from major international studies and using cutting-edge multi-omics technologies, the project seeks to enable more accurate, personalized risk stratification and treatment for patients with decompensated cirrhosis and ACLF. | Biomarkers and risk stratification |
| (76) | miRNA as biomarkers in early detection and personalized treatment in ovarian cancer (miRPOC) | Other chronic diseases | Secondary | To validate a miRNA panel for early detection of epithelial ovarian cancer (EOC) using serum samples from prospective cohorts. The goal is to develop a diagnostic tool that, with CA125, can improve early-stage EOC diagnosis and guide patients to personalized gyn-oncological care. The study will evaluate miRNA profiles for their ability to distinguish EOC from benign conditions and other cancers, and address ethical, legal, and social issues related to health data privacy and AI model development. | Screening and early diagnosis |
| (22) | Monitoring multidimensional aspects of QUAlity of Life after cancer ImmunoTherapy -  an Open smart digital Platform for personalized prevention and patient management (QUALITOP) | Cancer | Tertiary | To develop a European immunotherapy-specific open Smart Digital Platform using big data analysis, AI, and simulation modelling. This platform will collect and aggregate real-world data from clinical centers across four EU countries to monitor cancer patients’ health status and quality of life (QoL) post-immunotherapy. The project will provide “real-time” recommendations for care coordination and predict adverse events, ultimately leading to cost-effective guidelines for personalized cancer immunotherapy management. | Biomarkers and risk stratification |
| (37) | N6 - methyladenosine RNA modification in acute coronary syndrome (MAACS) | Cardiovascular diseases | Secondary | To use N6-methyladenosine (m6A) RNA modifications in PBMCs as a biomarker for acute coronary syndrome (ACS) diagnosis and prediction of adverse outcomes. The project will identify ACS-specific m6A signatures, quantify m6A modifications in PBMCs, and assess their clinical utility. | Biomarkers and risk stratification |
| (68) | Next generation tools for genome-centric multimodal data integration in personalized cardiovascular medicine (NextGen) | Cardiovascular diseases | N.d. | To address the fast-growing EU healthcare expenditure by developing tailored, accurate, and personalized interventions through the integration of multimodal data, including genomics data. It tackles challenges in data privacy, governance, and standardization, by providing tools for multimodal data integration, secure federated analytics, and scalable genomics data curation. NextGen’s comprehensive approach will enhance clinical efficiency, data discoverability, and management, aligned with initiatives like “1+ Million Genomes” and the European Health Data Space. Real-world pilots in the NextGen Pathfinder network will demonstrate the effectiveness of these tools. | Treatment personalization |
| (45) | Omics Approach for Personalized Prevention of Type 2 Diabetes Mellitus for African and European Populations (OPTIMA) | Metabolic diseases | Primary | To develop ethnic- and sex-specific algorithms for early prediction of dysglycemia, tailored to European and sub-Saharan African populations. Through transnational collaboration, the project integrates data from African (South African and Ghanaian) and European (Swedish) cohorts, combining proteomics and metabolomics to identify biomarkers specific to ethnicity and sex. The project aims to inform culturally acceptable, cost-effective dietary modifications for the prevention of type 2 diabetes, and assess their cost-effectiveness and cultural perceptions across different populations. | Biomarkers and risk stratification |
| (32) | PANcreatic CAncer Initial Detection via liquid biopsy (PANCAID) | Cancer | Secondary | To develop an accurate blood test for early detection of pancreatic cancer (PDAC). The project will analyze large patient cohorts, including those with early-stage PDAC, to identify effective biomarkers. It will establish a resource of blood samples, integrate multimodal features using AI, and evaluate socio-economic and ethical implications. The goal is to validate the test in a multi-center study, potentially enabling LBx screening for high-risk individuals as part of the Cancer Mission’s ‘Prevention’ initiatives. | Biomarkers and risk stratification |
| (33) | Personalized Cancer Medicine for all EU citizens (PCM4EU) | Cancer | Tertiary | To improve cancer patient outcomes in the EU through personalized cancer medicine (PCM). It seeks to address inequalities in access to PCM by implementing molecular diagnostics and drug repurposing trials across Europe. The consortium will establish best practices for genomics diagnostics, clinical decision support systems, and harmonization of molecular and clinical data interpretation. It will facilitate cross-border access to PCM, speed up patient enrolment in trials, and ensure the cost-effective integration of results into healthcare systems. | Treatment personalization |
| (40) | Personalizing the clinical decision making in ovarian cancer through patient-derived in vitro models (OVA-PDM) | Cancer | Secondary | To develop predictive biomarkers for personalized treatment with PARP inhibitors (PARPi) in high-grade serous ovarian cancer (HGSOC). Using patient-derived models like 3D organotypic cultures and organoids, the project aims to identify molecular pathways distinguishing responders from non-responders. Biomarkers will be validated in clinical cohorts, and personalized communication strategies will be developed to enhance patient well-being. | Biomarkers and risk stratification |
| (42) | Personalized CANcer Primary Prevention research through Citizen Participation and digitally enabled social innovation (4P-CAN) | Cancer | Primary | To develop personalized tools for primary cancer prevention in Central and Eastern European countries. Utilizing innovative approaches that combine social sciences, behavioral sciences, and technology with applied research, the project focuses on modifiable risk factors such as smoking, alcohol consumption, physical inactivity, overweight, HPV and HBV infections, and environmental pollution. The goal is to enhance policy implementation and individual adherence to healthy behaviors, thereby reducing inequalities and preventing cancer. | Implementation and participation |
| (21) | Personalized Prevention for Coronary Heart Disease (CoroPrevention) | Cardiovascular diseases | Secondary | To develop and implement a personalized secondary prevention program for patients with established Coronary Heart Disease (CHD). This strategy, tested in the CoroPrevention Trial, seeks to significantly reduce coronary events by using outcome- and patient-specific characteristics to guide prevention. Key objectives include evaluating the clinical utility and health economic benefits of personalized prevention, discovering predictive markers of drug response, refining ESC guidelines, and disseminating the program to healthcare practitioners, patients, payers, and policymakers across Europe. | Biomarkers and risk stratification |
| (57) | Prediction and prevention of cardiovascular diseases in pre- and type 2 diabetes (PROVIDE) | Cardiovascular diseases | Secondary | To develop a system for rapid screening of early CVD risk in diabetes patients, utilizing a combination of low-cost ECG technology, telehealth devices, advanced ECG analysis, and various biomarkers to predict individual cardiovascular risk. The secondary aim is to improve patient classification into sub-types of diabetes and differentiate comorbidities for better clinical decisions and personalized monitoring. The exploratory goal is to develop algorithms to create tailored diagnostic pathways and therapy strategies, moving away from the "one-size-fits-all" approach. | Biomarkers and risk stratification |
| (56) | Prediction of Alzheimer’s disease using an ai driven screening platform (PREDICTOM) | Neurological and psychiatric disorders | Secondary | To establish a scalable, cost-efficient diagnostic platform for early identification of Alzheimer’s disease (AD) and related disorders. This platform will utilize an open-source system and incorporate biomarkers from home-collected samples (blood, saliva, stool) along with innovative technologies like digital tools and biomarkers from imaging and blood tests. The platform aims to use AI models to identify individuals at high risk of developing dementia and guide them towards personalized interventions to prevent cognitive decline. | Biomarkers and risk stratification |
| (63) | Prediction, Monitoring and Personalized Recommendations for Prevention and Relief of Dementia and Frailty (COMFORTAGE) | Neurological and psychiatric disorders | N.d. | To create a pan-European framework for integrated, people-centric prevention, monitoring, and management of age-related diseases and disabilities. This framework combines medical innovations (personalized prediction, AI-based medical devices), cutting-edge AI technologies (explainable AI, Virtual Assistive technologies), Digital Innovation Hubs (Smart Homes, robotics), and social innovations for improved social integration. By integrating and harmonizing diverse data sources into a standardized structure called Holistic Health Records, the project will facilitate the prevention, monitoring, and management of diseases like dementia and frailty through advanced research and technological applications. | Biomarkers and risk stratification |
| (43) | Preventing lifetime obesity by early risk-factor identification, prognosis and intervention (eprObes) | Metabolic diseases | Primary | To identify and understand early life factors and mechanisms contributing to obesity, from prenatal to pubertal periods, including pathophysiological and psychological determinants. This multidisciplinary approach combines clinical studies, lifestyle and behavioral studies, and mechanistic analyses in preclinical models to define effective prevention strategies for obesity. By integrating multi-omics data and utilizing bioinformatic technologies and AI, the project aims to develop personalized preventive measures and interventions to manage body weight and prevent lifelong metabolic complications in both genders. | Treatment personalization |
| (12) | PREvention of STroke in Intracerebral hemorrhaGE survivors with Atrial Fibrillation (PRESTIGE-AF) | Cardiovascular diseases | Tertiary | To improve antithrombotic stroke prevention in intracerebral hemorrhage (ICH) patients with atrial fibrillation by: 1) conducting a robust randomized controlled trial (RCT) to test if direct oral anticoagulants (OAC) are superior for ischemic stroke (IS) prevention and non-inferior regarding ICH recurrence compared to antiplatelet therapy or no antithrombotic therapy; 2) developing personalized antithrombotic prevention strategies using multidimensional risk modeling, including clinical characteristics, magnetic resonance imaging (MRI), blood biomarkers, and genetics; 3) estimating the population-level impact of trial findings on health economics and generalizability to the European population; 4) exploring patient-centered factors such as adherence, attitudes toward antithrombotic therapy, and addressing gender imbalances in trial participation. | Biomarkers and risk stratification |
| (36) | Prodromal DEtErminants for PhENoconversion of idiopathic RBD to alpha-synucleinopathies (PD, DLB and MSA) (DEEPEN-iRBD) | Neurological and psychiatric disorders | Secondary, tertiary | To develop a proof of concept for predicting phenoconversion from idiopathic REM sleep behavior disorder (iRBD) to Parkinson’s disease (PD), dementia with Lewy bodies (DLB), or multiple system atrophy (MSA). This project integrates advanced clinical assessments, molecular markers, and patient samples to identify disease-specific profiles at the prodromal stage. By employing pathogenicity models and comprehensive genetic screening, the project seeks to provide early diagnosis, accurate prognosis, and personalized therapeutic interventions. The project also addresses ethical and social aspects related to communication and screening for individuals at risk in the prodromal stage, focusing on secondary and tertiary prevention. | Biomarkers and risk stratification |
| (41) | PRostate cancer Awareness and Initiative for Screening in the European Union (PRAISE-U) | Cancer | Secondary | To enhance Prostate Cancer (PCa) detection in EU member states through a cost-effective, individualized diagnostic strategy. The project involves assessing needs across member states, piloting a flexible risk-based screening algorithm, and aligning with the European Guidelines for Quality Assurance in Breast Cancer Screening and Diagnosis. It brings together key experts from various disciplines to enable knowledge transfer and design national screening strategies. | Screening and early diagnosis |
| (62) | Real-world implementation, deployment and validation of early detection tools and lifestyle enhancement (AD-RIDDLE) | Neurological and psychiatric disorders | Primary, secondary, tertiary | To address Alzheimer's disease (AD) by developing a comprehensive toolbox platform that includes community outreach tools, clinically validated biomarkers, digital cognitive tests, and validated algorithms for early detection and prevention. This platform will enable healthcare systems and practitioners to implement enhanced AD management across diverse patient populations, offering potential for cost-effective preventive therapies. The project aims to revolutionize AD detection, diagnosis, and treatment, with scalability for other dementias, thereby reducing the burden on healthcare systems and improving public health outcomes. | Biomarkers and risk stratification |
| (71) | Residual disease assessment in hematologic malignancies to improve patient-relevant outcomes across Europe (RESOLVE) | Cancer | Tertiary | To establish measurable residual disease (MRD) detected by multiparameter flow cytometry (MFC) as a predictive biomarker for guiding treatment in patients with acute myeloid leukemia (AML) and chronic lymphocytic leukemia (CLL). The project will utilize existing networks to develop a patient registry and standardized MRD analysis across Europe, along with a multinational trial to test the hypothesis that treatment intensity can safely be reduced for MRD negative patients. This project aims to improve clinical decision-making and access to MRD testing for all AML and CLL patients, addressing a major unmet need in cancer treatment. | Biomarkers and risk stratification |
| (25) | Revision and update of the European Code against Cancer (ECAC5) | Cancer | Primary | To develop the 5th edition of the European Code against Cancer (ECAC) by updating it based on the latest scientific evidence and expanding prevention measures to target different audiences. This initiative aligns with the Europe Beating’s Cancer Plan and incorporates a multidisciplinary approach, integrating behavioral science, technological innovations, and omics-based insights to enhance cancer prevention literacy. Building on the setup of previous editions, ECAC5 seeks to provide a unified EU strategy for cancer prevention, offering evidence-based recommendations and promoting effective communication to influence national health policies. | Other |
| (73) | SPatial Analysis of Cancer Evolution in the Tumor Immune MicroEnvironment (SPACETIME) | Cancer | Tertiary | to better understand and overcome resistance to immune checkpoint therapy in Non-Small Cell Lung Cancer (NSCLC) by exploring the tumor microenvironment. Using advanced omics technologies, including spatial proteomics, transcriptomics, metabolomics, and glycomics, the project seeks to identify biomarkers and mechanisms driving therapy resistance. The goal is to develop a spatial signature test for patient stratification, leading to more effective, personalized treatments and improved survival outcomes for NSCLC patients. | Biomarkers and risk stratification |
| (19) | Specific Imaging of Immune Cell Dynamics Using Novel Tracer Strategies (Immune-Image) | Cancer | Tertiary | To revolutionize the measurement and monitoring of immune status through the development of new immunotracers for non-invasive whole-body imaging techniques, such as PET, MRI, and Optical Imaging. This approach seeks to enhance our understanding of patient immune responses and support personalized immunotherapy. The project involves an international consortium of experts across multiple disciplines, including chemistry, biology, immunology, physics, pharmacy, and information technology. By providing insights into immune drug use and improving immunotherapy development, Immune-Image aims to refine patient care and treatment decisions, ultimately leading to more effective therapies for immune-related diseases. | Treatment personalization |
| (60) | Targeting tumor-host interactions in pediatric malignant gliomas to reinvigorate immunity and improve radio- and immunotherapy efficacy (HIT-GLIO) | Cancer | Tertiary | To address the challenges of pediatric high-grade gliomas (pHGGs), which are malignant and difficult to treat due to their aggressive nature and immunosuppressive tumor microenvironment (TME). The project focuses on understanding tumor-host interactions in the TME to identify new therapeutic targets. HIT-GLIO will utilize advanced technologies like single-cell analysis and multimodal imaging, as well as create complex models such as human glioma-microglia co-cultures and pHGG-derived organoids, to study these interactions. It will also explore hypoxia-inducible epigenetic inhibitors, nanocarriers, and reprogramming strategies for immune cells to enhance the efficacy of radiotherapy (RT) and immunotherapy. | Treatment personalization |
| (17) | The Brain Health Toolbox: Facilitating personalized decision-making for effective dementia prevention (Brain Health Toolbox) | Neurological and psychiatric disorders | N.d. | To address the global challenge of preventing dementia and Alzheimer’s disease (AD) through a new multimodal approach. The project focuses on developing disease models and prediction tools that incorporate a broad range of risk factors and biomarkers, including novel markers, to create a seamless continuum from accurate dementia prediction to effective prevention. An innovative machine learning method will be used for risk profiling to identify individuals at high risk and monitor their disease progression. The Toolbox will be tested in multimodal prevention trials across different geographic, economic, and cultural settings, leveraging reference libraries from both observational and interventional studies. | Biomarkers and risk stratification |
| (74) | The European Comprehensive Cancer Centre Network (EUnetCCC JA) | Cancer | Primary, secondary, tertiary | To create a cohesive and integrated consortium of Comprehensive Cancer Centers (CCCs) across Europe. The primary objective is to ensure that all patients, regardless of location, have access to high-quality care. The network will foster collaboration among centers to share best practices, resources, and knowledge, enhancing research, clinical care, and treatment methods. It will address inequalities in diagnosis, treatment, and access to clinical trials, while integrating clinical care with research to improve cancer care quality throughout Europe. This collaboration will ultimately benefit patients by improving access to innovative treatments and diagnostics. | Screening and early diagnosis |
| (78) | Tumor-host interactions in liver cancer of childhood and adults (THRIVE) | Cancer | Secondary, tertiary | To improve outcomes for liver cancer patients by: 1) understanding at-risk populations and tumor-host interactions; 2) developing biomarkers for therapies; 3) creating a comprehensive human liver cancer blueprint; 4) identifying markers for treatment response; 5) implementing a preclinical drug testing platform; 6) providing accessible, re-usable data to support EU initiatives and influence health policies. | Biomarkers and risk stratification |
| (48) | Urine biomarkers for bladder cancer diagnosis and surveillance: a multicentric study to assess the diagnostic accuracy of a comprehensive diagnostic tool (UBIOBCA) | Cancer | Secondary | To develop non-invasive, cost-effective urine-based diagnostic tests for early detection and monitoring of bladder cancer (BC). Given the limitations of current invasive diagnostic methods, these new biomarkers aim to provide a sensitive and universally applicable alternative. Through a large international study involving Canada, France, and Germany, the project will assess the diagnostic accuracy of these tests, both individually and in combination, to improve detection of primary and recurrent BC. The collaboration of experts from multiple countries will facilitate the clinical validation and potential global implementation of these tests. | Biomarkers and risk stratification |
| (61) | Well-being improvement through the Integration of healthcare and reSearch Data and models with Out border for chronic iMmune-mediated diseases (WISDOM) | Other chronic diseases | Tertiary | To integrate and utilize medical and research data using advanced AI to transform biological information into actionable insights for chronic immune-mediated diseases (CIMDs). The consortium, which includes leading European institutions and organizations, seeks to address challenges in data accessibility and integration. The goal is to develop AI-driven tools for risk stratification and outcome prediction, facilitating patient-centered diagnosis and treatment. WISDOM aims to promote the responsible use of AI in healthcare through collaboration among clinicians, researchers, and stakeholders, ultimately revolutionizing health data management for CIMDs. | Biomarkers and risk stratification |
